# Compressing Semantic Segmentation Models for Agricultural IoT: A Systematic Study of Pruning Trade-Offs for Crop–Weed Segmentation

**DOI:** 10.3390/jimaging12100495

**Published:** 2026-10-08

**Authors:** Flora Amato, Rajib Chandra Ghosh, Semanto Mondal

**Affiliations:** Department of Electrical Engineering and Information Technology (DIETI), University of Naples Federico II, 80125 Naples, Italy; flora.amato@unina.it (F.A.); semanto.mondal@unina.it (S.M.)

**Keywords:** semantic segmentation, network pruning, model compression, precision agriculture

## Abstract

Semantic segmentation of crops and weeds is an important component of selective weeding systems, but accurate segmentation networks can impose substantial computational and memory demands on resource-constrained agricultural platforms. This paper presents a systematic study of the accuracy–efficiency trade-offs involved in compressing semantic segmentation networks for agricultural edge computing. Six architectures spanning conventional CNN encoder–decoders, atrous and pyramid-pooling decoders, a mobile-oriented CNN, and two transformer scales are evaluated on the Crop/Weed Field Image Dataset (CWFID), with three random seeds per architecture. The most favourable compact model is subsequently evaluated under unstructured global magnitude pruning and dependency-aware structured channel pruning, using one-shot and iterative prune-and-fine-tune schedules at sparsity levels from 10% to 80%, with three seeds per configuration. Among the dense baselines, U-Net with a MobileNetV3-Large encoder matches the accuracy of the larger SegFormer-B2 model (88.85% versus 88.67% mIoU, a difference of 0.18% across three seeds, *p* = 0.58) with 3.7× fewer parameters than SegFormer-B2. Iterative unstructured pruning reaches 80% weight sparsity while retaining 89.04% mIoU; however, because the dense tensor dimensions are unchanged, this sparsity does not reduce the parameter count, GFLOPs, or model size on conventional hardware. Structured channel pruning, which does reduce all three, remains viable only up to 30% sparsity, reducing GFLOPs by 2.1× at a cost of 4.21 mIoU points and collapsing beyond 40%. Without fine-tuning, structured pruning causes severe degradation even at 10% channel sparsity. The results show that architecture selection has a major influence on the accuracy–efficiency trade-off, while the effects of pruning depend strongly on the pruning structure and fine-tuning strategy. Crop segmentation consistently degrades before the aggregate mIoU indicates severe failure, highlighting the need for class-wise evaluation when compressing crop–weed segmentation models.

## 1. Introduction

### 1.1. Semantic Segmentation for Crop–Weed Discrimination

Selective weed management is an important application of computer vision in precision agriculture, where the objective is to identify weeds and target them without unnecessarily affecting crop plants. Deep learning has increasingly replaced hand-crafted image features in this setting, with semantic segmentation being particularly useful because it provides pixel-level localization of crop and weed regions rather than only image- or object-level predictions [1,2,3]. This spatially detailed perception is valuable for robotic and site-specific weeding systems, but it also places a substantial computational burden on the perception module: segmentation networks must process high-resolution images while operating within the latency, memory, and power constraints of an on-board computing platform. Recent reviews of real-time agricultural vision similarly identify the limited adoption of edge deployment and the need to balance segmentation accuracy with computational efficiency as persistent challenges in practical weed-management systems [3,4], and recent studies have proposed increasingly lightweight networks for on-board crop–weed segmentation [5,6,7].

The Crop/Weed Field Image Dataset (CWFID), introduced by Haug and Ostermann [8], has become a commonly used benchmark for this setting. The dataset contains field images acquired by an autonomous agricultural robot and includes closely spaced carrot plants and weeds, making crop–weed discrimination particularly challenging. Subsequent studies have applied convolutional segmentation networks to this and related agricultural datasets. For example, Fathipoor et al. [9] compared U-Net and U-Net++ for crop–weed segmentation and reported improved detection of small weeds with U-Net++. Su et al. [10] investigated data augmentation for crop–weed segmentation in agricultural robotics, showing that augmentation can improve segmentation performance when training data are limited. More recently, lightweight feature-fusion architectures have also been explored for crop–weed segmentation; for example, the multi-level feature re-weighted fusion network of Janneh et al. [11] was evaluated on the CWFID together with other agricultural datasets and was designed to improve segmentation accuracy while reducing computational requirements. These studies demonstrate the effectiveness of deep segmentation models for crop–weed discrimination, but they primarily focus on proposing or evaluating individual architectures or task-specific improvements.

The development of semantic segmentation has produced increasingly capable convolutional and transformer-based architectures. Encoder–decoder networks such as U-Net remain effective for dense prediction because their skip connections preserve spatial details that are important for delineating small and thin structures [12]. In parallel, lightweight architectures such as MobileNetV3 were explicitly designed for resource-constrained platforms through hardware-aware architecture optimization [13]. More recently, transformer-based segmentation models such as SegFormer have demonstrated that strong segmentation accuracy can also be achieved with hierarchical attention-based representations and efficient decoder designs [14]. In agricultural applications, recent work has similarly emphasized real-time or edge deployment: Rai et al. [15] investigated an edge-oriented weed-identification system with an emphasis on computationally efficient deep learning. These developments, however, do not imply that a larger or newer architecture is necessarily the best choice for a constrained agricultural platform. In practice, the relevant question is not only which model is most accurate, but which model provides the most favorable balance among segmentation quality, parameter count, computational cost, and deployability. Moreover, comparisons across architectures are difficult when models are trained under different data splits, preprocessing pipelines, augmentation strategies, optimization settings, or evaluation protocols; reported performance values from separate studies should therefore not be interpreted as direct evidence that one architecture is universally superior to another.

### 1.2. Model Compression for Dense Prediction

Model compression provides a complementary route to reducing this computational burden. Network pruning removes parameters or structural components that are considered less important, with early magnitude-based methods demonstrating that substantial portions of trained networks can be removed while retaining useful predictive performance [16,17]. Iterative magnitude pruning further demonstrated that high levels of weight sparsity can sometimes be obtained while retaining the predictive capability of the original dense network [18]. Pruning strategies can broadly be divided into unstructured pruning, which removes individual weights, and structured pruning, which removes groups such as filters or channels. Unstructured pruning can achieve high sparsity but preserves the original tensor dimensions and therefore does not automatically translate into lower dense-runtime latency. Structured pruning produces physically smaller networks that can be executed using standard dense operators and can therefore provide more direct computational savings; representative approaches include filter pruning such as ThiNet [19] and channel-level network slimming [20]. However, removing entire channels is more disruptive, requires careful handling of dependencies between layers [21], and is more difficult to obtain without sacrificing accuracy than unstructured sparsity [22]. The pruning literature has also highlighted substantial inconsistencies in evaluation protocols, making comparisons across pruning studies difficult when datasets, seeds, metrics, fine-tuning procedures, and measures of computational cost are not controlled consistently [23]; recent surveys of pruning for edge deployment reiterate this point [24].

This distinction is particularly important for semantic segmentation. Unlike image classification, segmentation requires spatially detailed predictions, and removing filters or channels can affect the representation of fine structures and object boundaries. Recent pruning research has therefore emphasized that methods developed primarily for classification cannot necessarily be transferred to segmentation without considering its spatial structure. For example, Wu et al. [25] explicitly argue that conventional filter-pruning strategies may overlook the location-sensitive nature of semantic segmentation and propose a spatially aware structural pruning strategy, and further segmentation-specific pruning criteria have been proposed recently [26]. Such findings motivate evaluating pruning directly at the segmentation-task level rather than assuming that compression behavior observed in classification will transfer unchanged to dense prediction.

These issues are particularly relevant to agricultural semantic segmentation. Most crop–weed studies focus primarily on segmentation accuracy, while comparatively less attention is given to how different architectures behave under systematic compression and how compression affects individual semantic classes. This is important because crop and weed segmentation is not a symmetric classification problem: a model can retain a reasonable overall score while increasingly confusing crop pixels with weeds. Similarly, a high percentage of zero-valued weights does not necessarily correspond to a proportional reduction in inference cost, whereas structured channel removal can reduce the actual dimensions of the network but may cause a much larger accuracy loss. Thus, compression decisions for agricultural vision cannot be based on sparsity or mean segmentation accuracy alone. A recent review of real-time deep learning segmentation for precision weed management likewise identifies pruning, quantization, lightweight architectures, and edge deployment as emerging directions, while noting that real-world deployment and the relationship between computational reduction and practical inference remain insufficiently characterized [4].

### 1.3. Research Gap, Questions, and Contributions

Despite these developments, the existing literature leaves an important evaluation gap for crop–weed segmentation. Agricultural studies have demonstrated increasingly accurate or lightweight segmentation architectures, and pruning studies have established general principles for reducing neural-network redundancy, but these lines of work are rarely examined together under a common experimental protocol. In particular, there is limited systematic evidence comparing different segmentation architectures first and then examining how both unstructured weight sparsity and structured channel removal affect accuracy, computational cost, and class-wise segmentation quality on the same agricultural task. The influence of the pruning schedule is also important because one-shot removal and progressive pruning followed by fine-tuning can lead to substantially different outcomes, yet this factor is not consistently examined in agricultural segmentation studies.

In this work, we conduct a systematic study of these trade-offs using the CWFID [8]. Six segmentation architectures covering conventional CNN encoder–decoders, atrous and pyramid-pooling designs, a mobile-oriented CNN, and two transformer scales are evaluated under a common training and test protocol with three random seeds. The most favourable compact architecture is then subjected to controlled unstructured and structured pruning experiments over sparsity levels from 10% to 80%, with both one-shot and iterative prune-and-fine-tune schedules. In addition to mean IoU, we explicitly track per-class IoU, parameter counts, non-zero parameters, GFLOPs, checkpoint size, and measured inference latency. The study is deliberately scoped as a controlled, single-dataset analysis: its purpose is to isolate the effects of architecture, pruning mode, and pruning schedule under identical conditions, not to establish results that transfer to other crops or hardware, and the conclusions are stated with that scope. Importantly, the objective is not to establish a direct ranking against numerical results reported in individual prior studies; differences in datasets, train–test partitions, preprocessing, augmentation, model initialization, hardware, and optimization procedures can substantially affect reported results across papers. Instead, the present work places the evaluated architectures and pruning strategies under the same experimental conditions, thereby providing a controlled basis for analyzing their relative accuracy–efficiency and compression trade-offs. The study is guided by the following research questions:

- RQ1: Which segmentation architecture provides the most favorable accuracy–efficiency trade-off for crop–weed segmentation under a common training and evaluation protocol?
- RQ2: How do unstructured weight pruning and structured channel pruning affect segmentation accuracy and computational complexity as sparsity increases?
- RQ3: How do pruning schedules and fine-tuning influence compression robustness, and which semantic classes degrade first under increasingly aggressive pruning?

The main contributions of this study are as follows:

- We provide a seed-controlled comparison of six representative semantic segmentation architectures on the CWFID, jointly evaluating segmentation quality and model complexity. The experiments show that a U-Net with a MobileNetV3-Large encoder achieves accuracy that is not statistically distinguishable from the best transformer model under the evaluated three-seed protocol while requiring substantially fewer parameters than the largest competing models.
- We perform a controlled comparison of unstructured global magnitude pruning and dependency-aware structured channel pruning across eight sparsity levels and two pruning schedules. The results demonstrate a pronounced difference between the two compression mechanisms: iterative unstructured pruning retains the baseline performance even at 80% weight sparsity, whereas structured pruning provides direct reductions in parameters and GFLOPs but remains reliable only at substantially lower sparsity.
- We examine the class-wise effects of compression with per-class IoU for all three classes, confusion matrices and colour-coded error maps, and show that crop segmentation degrades before the aggregate mIoU indicates severe failure. This demonstrates the importance of reporting per-class segmentation performance when evaluating compressed crop–weed models.
- We provide practical guidance for selecting and compressing segmentation models under resource constraints, while explicitly distinguishing weight sparsity from reductions in the computational graph, supported by measured inference latency.

The remainder of the paper is organised as follows. Section 2 describes the dataset, the evaluated architectures, the training and pruning protocols, and the evaluation metrics. Section 3 reports the experimental results, Section 4 interprets them with respect to the research questions and discusses limitations, and Section 5 concludes the paper.

## 2. Materials and Methods

**Figure 1:**
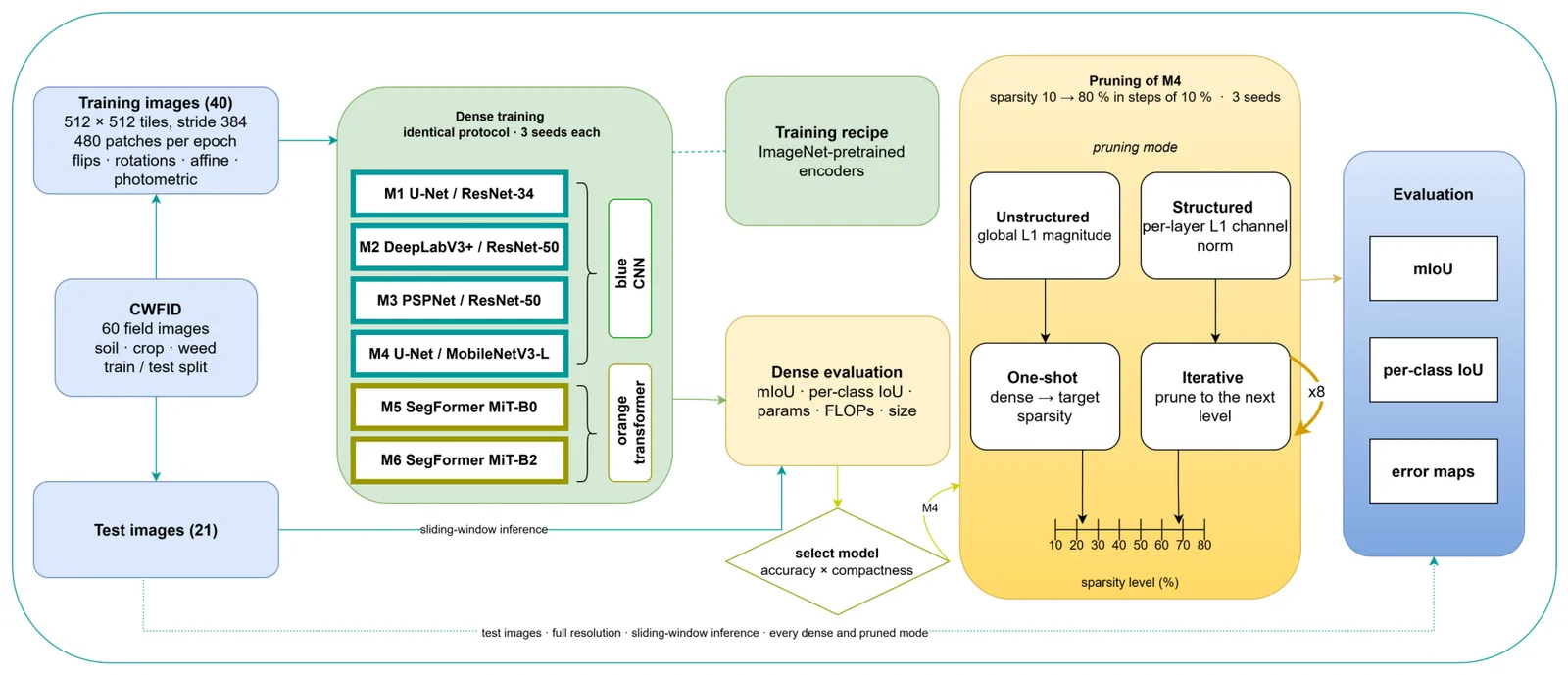
Overview of the experimental pipeline: dataset partitioning and pre-processing, seed-controlled training of six segmentation architectures, model selection, the four pruning configurations at eight sparsity levels, and the evaluation metrics.

Figure 1 summarises the experimental pipeline. First, the Crop/Weed Field Image Dataset is split into tiles, augmented, and the original train-test split is used so that all numbers are based on field images. Second, six ImageNet-pretrained segmentation models from both convolutional and transformer families are trained according to a unified seed-based training protocol, and the best model is chosen for compression. Third, the chosen model is compressed using four pruning configurations by combining two pruning modes (unstructured and structured) with two schedules (one-shot and iterative). The performance of the pruned models is evaluated by IoU, per-class IoU, F1, and four cost metrics: total and non-zero parameters, GFLOPs, checkpoint size, and inference latency.

### 2.1. Dataset and Pre-Processing

**Figure 2:**
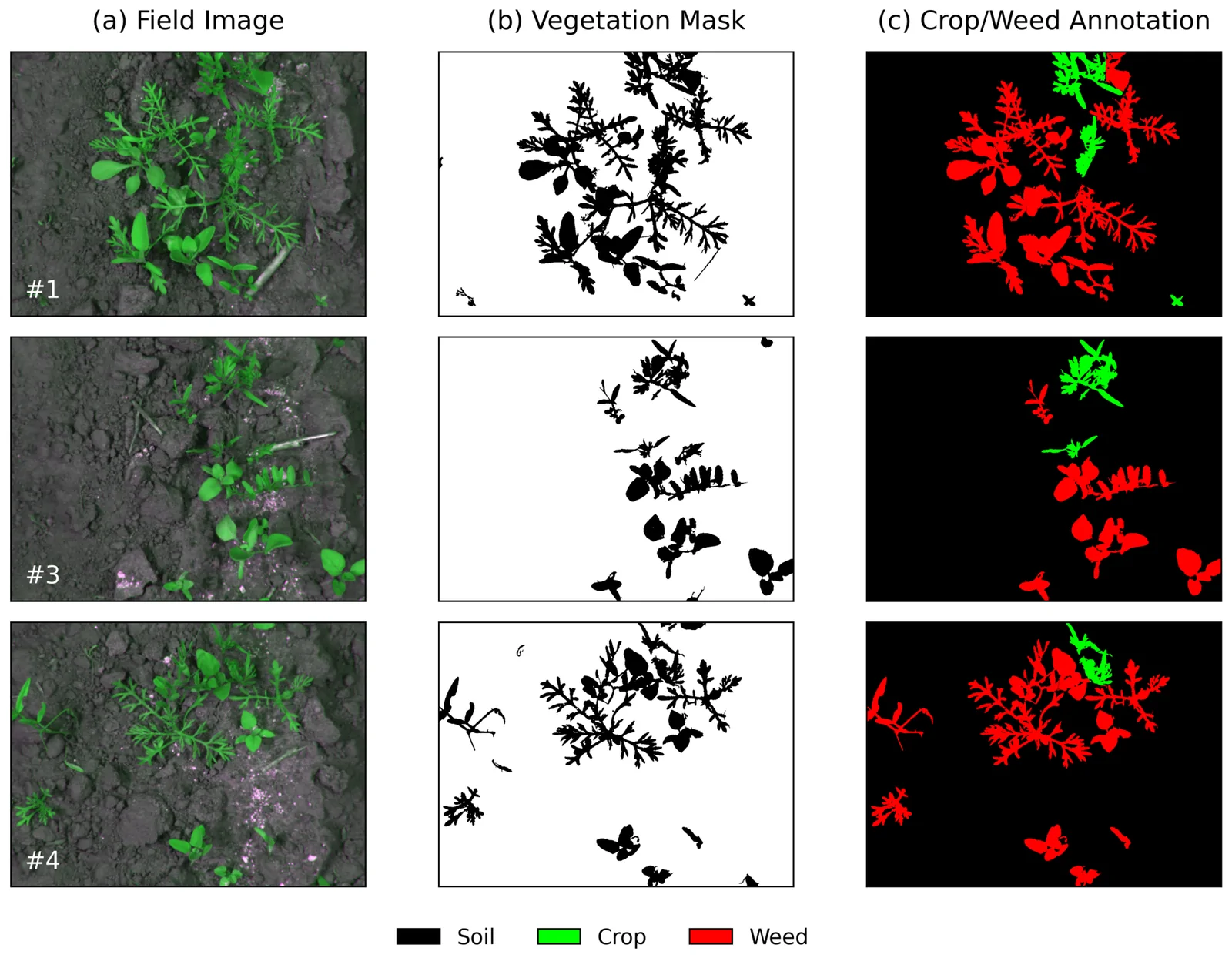
Representative CWFID samples. (**a**) Field images acquired by the BoniRob platform; (**b**) binary vegetation masks; (**c**) pixel-level annotations, where black denotes soil, green denotes crop, and red denotes weed.

This study employs the Crop/Weed Field Image Dataset (CWFID) [8], a publicly available benchmark collected using the BoniRob field robot in an organic carrot field under natural illumination. The dataset contains 60 RGB images with a spatial resolution of 1296×966 pixels, accompanied by pixel-level annotations for three semantic classes: soil, crop (carrot), and weed. CWFID was selected due to its established use as a benchmark for crop–weed segmentation in agricultural robotics [1,2,27], as well as the availability of a pre-defined train/test partition that facilitates reproducible comparisons across segmentation architectures and model-compression strategies. Figure 2 presents representative CWFID samples, illustrating the original field images, corresponding vegetation masks, and pixel-level crop/weed annotations. In the test images, soil occupies 92.6% of the pixels, weed 5.7%, and crop 1.7%, so the two plant classes are strongly underrepresented, and the crop class is the rarest.

The official dataset partition was retained without modification, comprising 40 training images and 21 test images. The test set was not used for model training, hyperparameter tuning, or checkpoint selection. The test split is only used at the final evaluation and for the selection of the architecture carried forward to the pruning study, which is stated explicitly in Section 3.1. During training, the images were divided into overlapping 512×512-pixel patches using a stride of 384 pixels, with an additional patch anchored at the bottom and right image borders so that every pixel is covered, resulting in 12 patches per image and 480 training patches per epoch. To improve robustness to spatial and photometric variability, we applied data augmentation using random horizontal and vertical flips, 90° rotations, small affine perturbations, and photometric transformations involving brightness, contrast, and hue.

At inference, each full-resolution image is covered by 512×512 windows with a stride of 384 pixels along both axes, identical to the training tiling, with an additional window anchored at the far edges so that every pixel is covered. The identical procedure is applied to every dense and pruned model, so all reported metrics are computed on complete 1296×966 field images and are directly comparable.

### 2.2. Segmentation Architectures

Semantic segmentation assigns a class label to every pixel of an image. For selective weeding, this is the appropriate formulation because the actuator must know not only whether a weed is present but where its leaves and stem lie relative to the neighbouring crop plants, which image-level classification or bounding-box detection cannot provide. Modern segmentation networks consist of an encoder, usually an image-classification backbone that produces progressively coarser feature maps, and a decoder that restores full-resolution predictions from those features. The design of the decoder determines how well thin structures and small objects are preserved, which is decisive for CWFID, where crops and weeds are only a few pixels wide.

To investigate whether the accuracy–efficiency trade-off varies across architectural families and model capacities, we evaluate six ImageNet-1K-pretrained [28] semantic segmentation networks representing both convolutional neural networks (CNNs) and hierarchical vision transformers. The selected architectures encompass a range of encoder–decoder designs and model capacities relevant to resource-constrained deployment. Specifically, three conventional CNN-based encoder–decoder architectures are considered: U-Net [12] with a ResNet-34 encoder [29] (M1), DeepLabV3+ [30] with a ResNet-50 encoder (M2), and PSPNet [31] with a ResNet-50 encoder (M3). These models represent, respectively, skip-connection-based decoding, atrous spatial processing, and pyramid-pooling-based feature aggregation; the latter two predict at a reduced output stride and recover resolution by upsampling, whereas U-Net fuses high-resolution encoder features through skip connections. To further examine lightweight architectures, U-Net with a MobileNetV3-Large encoder [13] (M4) is included as a mobile-oriented CNN configuration. Finally, two SegFormer [14] variants with MiT-B0 (M5) and MiT-B2 (M6) encoders are considered to represent hierarchical transformer-based segmentation architectures with different capacity levels. The six models were chosen so that each of the following design factors is represented by at least one pair that differs only in that factor: decoder type at equal encoder (M2 versus M3), encoder size at equal decoder (M1 versus M4, M5 versus M6), and CNN versus transformer at comparable size (M4 versus M5, M1 versus M6). Overall, the selected models span a parameter range from 3.7 to 26.7 M and a computational complexity from 13.5 to 73.8 GFLOPs for a 512×512 input, thereby providing a diverse basis for analyzing the relationship between segmentation accuracy, computational cost, and model capacity. The complete list of evaluated architectures and their complexity is provided in Table 1.

### 2.3. Training Protocol

All experiments were conducted under a unified experimental protocol to ensure a consistent and controlled comparison across architectures and pruning configurations. All dense models were trained for 60 epochs using a batch size of 8. Each model was optimized using AdamW with a weight decay of $1\times 10^{-4}$. The learning rate was governed by a one-cycle schedule with a maximum learning rate of $3\times 10^{-4}$ and a warm-up phase corresponding to 10% of the training schedule. The training objective was defined as the sum of the cross-entropy and Dice losses, providing complementary optimization criteria for pixel-wise classification while helping mitigate class imbalance among the soil, crop, and weed categories. Automatic mixed precision (AMP) was used throughout training to reduce GPU memory consumption and improve computational efficiency.

The final checkpoint obtained at the end of each 60-epoch training run was evaluated once on the official test split. All quantitative results are reported as the mean ± standard deviation over three independent random seeds (0, 1, and 2). Training was performed on a system equipped with an Intel Core Ultra 9 processor, 32 GB of DDR5 RAM, and an NVIDIA GeForce RTX 5080 Laptop GPU with 16 GB of VRAM. The complete training and pruning configurations are summarized in Table 2.

### 2.4. Pruning Protocol

The dense baseline selected in Section 3.1 was evaluated at eight target sparsity levels, ranging from 10% to 80% in increments of 10%. Here, sparsity is defined as the fraction of removable parameters eliminated during pruning, corresponding to individual weights for unstructured pruning and output channels for structured pruning. At each sparsity level, four pruning configurations were evaluated by combining two pruning modes with two pruning schedules: (i) unstructured one-shot pruning, (ii) unstructured iterative pruning, (iii) structured one-shot pruning, and (iv) structured iterative pruning. Each pruning configuration was initialized independently from each of three independently trained dense-model seeds. Consequently, every reported pruning result represents the mean performance of three independently derived pruned networks, thereby reducing the influence of initialization variability. For all pruning experiments, the same loss function, optimizer, batch size, input resolution, data augmentation, and evaluation procedure used during dense training were retained. The stem and final prediction layers were excluded from pruning to preserve the input and output dimensions required by the segmentation task.

#### 2.4.1. Pruning Modes

Unstructured pruning employs global L1-magnitude pruning [16] across all prunable convolutional and linear weights. The target sparsity therefore denotes the proportion of these weights that are set to zero. Pruned weights are retained explicitly as zero-valued entries; consequently, unstructured pruning does not alter the network architecture, parameter count, nominal GFLOPs, or checkpoint size, although it reduces the number of non-zero weights.

Structured pruning removes output channels with the smallest L1 norm from each prunable layer using the dependency-graph-based pruner implemented in Torch-Pruning [21]. The dependency graph propagates channel removals to all affected operations and coupled layers, including subsequent convolutions, batch normalisation layers, residual additions, squeeze-and-excitation gates, and decoder concatenations. In the structured setting, the target sparsity is applied uniformly and independently to each eligible layer without rounding the resulting channel count to hardware-specific multiples. Uniform per-layer sparsity was chosen deliberately as the simplest and most reproducible baseline and so that the sparsity axis is directly comparable between the two pruning modes; layer-sensitivity analysis and global channel budgets are discussed as extensions in Section 4.4. Hardware alignment was not applied because it was tested in preliminary experiments and enforcing a multiple-of-eight constraint transformed a nominal 10% target into an approximately 50% reduction for the 16-channel layers of the mobile encoder, destroying the network at the first level. A consequence of this choice is that the structurally pruned models are not aligned to hardware-friendly channel multiples, which limits the acceleration that can be realised in practice (Section 3.2.4).

#### 2.4.2. Pruning Schedules

Two pruning schedules are considered. In the one-shot setting, weights or channels are removed directly from the dense model in a single operation to reach the target sparsity, without subsequent fine-tuning. This setting quantifies the immediate degradation introduced by pruning and provides a baseline for assessing the benefit of fine-tuning. In the iterative setting, pruning is performed sequentially across the predefined sparsity levels, and the model at level $s_{t}$ is obtained by pruning the fine-tuned model of level $s_{t-1}$ rather than the dense model. After each pruning step, the resulting model is fine-tuned for 10 epochs using AdamW under a one-cycle learning-rate schedule with a peak of $1\times 10^{-4}$ and a 10% warm-up, i.e., the dense-training schedule with a lower peak learning rate, and the same cross-entropy and Dice loss. The fine-tuning budget was fixed a priori and applied consistently across all pruning strategies and sparsity levels. Thus, the model evaluated at each sparsity level incorporates cumulative pruning and fine-tuning from all preceding levels, corresponding to a total of 10–80 fine-tuning epochs across the 10% to 80% sparsity range. It follows that the one-shot and iterative arms differ in two respects at once: the granularity of parameter removal and the amount of recovery training. The iterative model evaluated at sparsity $s_{t}$ has received 10t epochs of fine-tuning that the one-shot model at the same sparsity has not, up to 80 additional epochs at 80%. The comparison between the two arms therefore measures the combined effect of gradual pruning and of fine-tuning, and cannot isolate the contribution of gradual removal alone; a one-shot arm with matched fine-tuning would be required for that, and was not run.

### 2.5. Evaluation Metrics

Segmentation performance is evaluated using a single confusion matrix accumulated over all pixels from the test images. For each class *c*, the intersection-over-union (IoU) and F1-score are defined as

(1) $${\mathrm{IoU}}_{c}=\frac{TP_{c}}{TP_{c}+FP_{c}+FN_{c}}$$

(2) $$F1_{c}=\frac{2\;TP_{c}}{2\;TP_{c}+FP_{c}+FN_{c}}$$

where $TP_{c}$, $FP_{c}$, and $FN_{c}$ denote the numbers of true-positive, false-positive, and false-negative pixels, respectively, for class *c*. With C=3 classes (soil, crop, and weed), the mean IoU (mIoU) and mean F1-score (mF1) are computed as unweighted averages over all classes:

(3) $$\mathrm{mIoU}=\frac{1}{C}\sum_{c=1}^{C}{\mathrm{IoU}}_{c},\;\mathrm{mF}1=\frac{1}{C}\sum_{c=1}^{C}F1_{c}$$

Although mIoU provides a balanced measure across the three classes, aggregate metrics can be strongly influenced by the dominant soil class, which occupies a large proportion of the images and is generally easier to segment than the vegetation classes. To obtain a more informative measure of pruning-induced degradation, we report per-class IoU for all three classes for both the dense and the pruned models, treat crop IoU as the primary indicator of degradation because misclassifying a crop plant is the costlier error for a weeding system, and complement the aggregate metrics with row-normalised confusion matrices and colour-coded error maps that distinguish crop-labelled-as-weed, weed-labelled-as-crop, missed vegetation and false vegetation.

## 3. Results

### 3.1. Dense Architecture Comparison

#### 3.1.1. Overall Accuracy

Table 3 summarizes the performance of the six dense segmentation models on the CWFID test split. The results show substantial differences in the accuracy–efficiency trade-off among the evaluated architectures.

**Figure 3:**
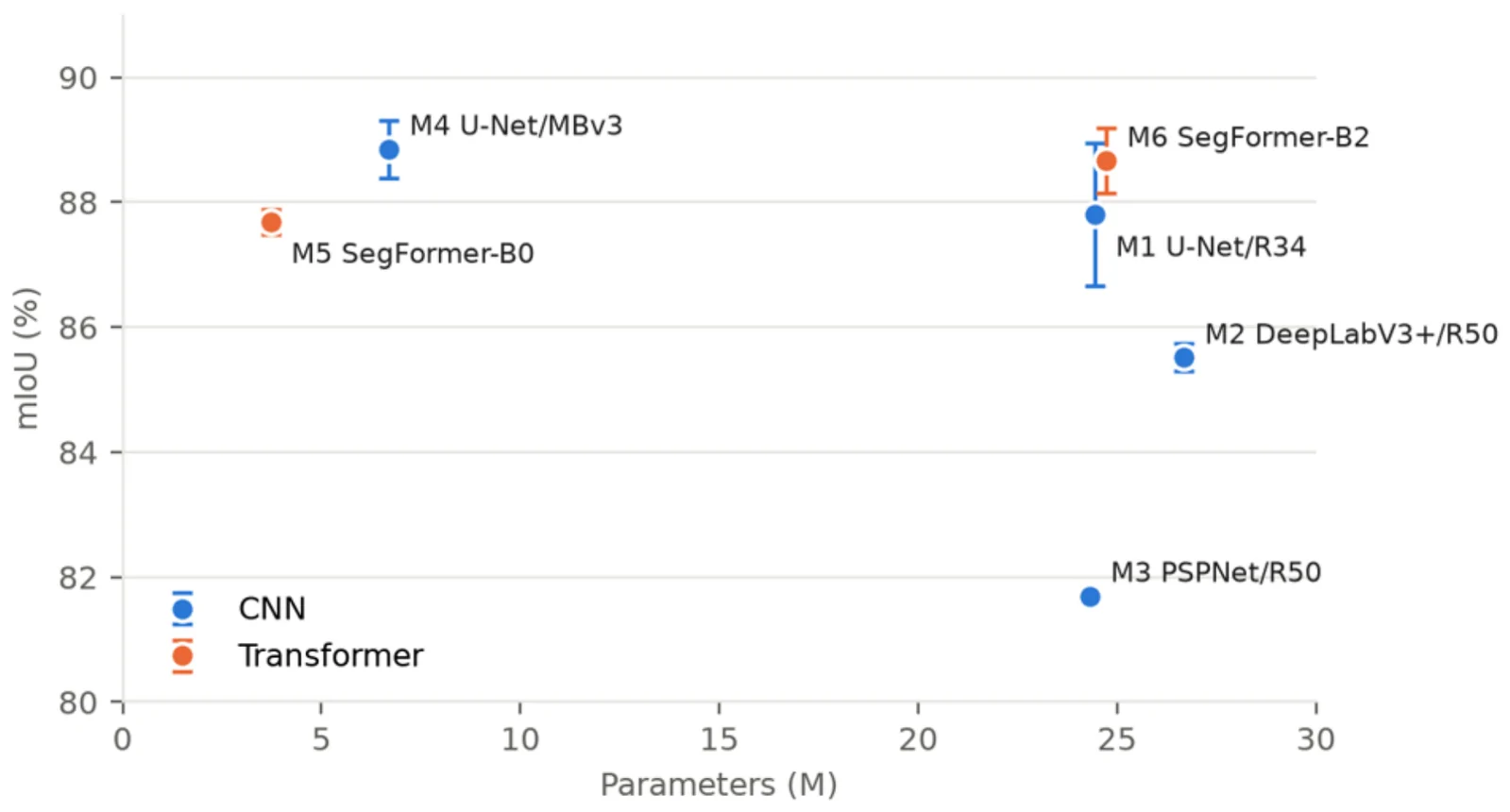
Mean IoU versus parameter count for the six dense segmentation baselines. Error bars indicate one standard deviation over three seeds.

The U-Net with a MobileNetV3-Large encoder (M4) achieves the highest mIoU, reaching 88.85±0.46%, where SegFormer-B2 (M6) obtains a similar mIoU of 88.67±0.53%, the difference is about 0.18 points (paired *t*-test, p=0.58, Table 4), but uses approximately 3.7× more parameters and 1.7× more GFLOPs than M4. At the other end of the model-size range, SegFormer-B0 (M5) contains only 3.71 M parameters and requires a 15.0 MB checkpoint, while achieving 87.70±0.20% mIoU, only 1.15 percentage points below M4 (p=0.08). Thus, higher parameter count does not consistently translate into higher segmentation accuracy for this dataset. The three ResNet-based models, despite containing approximately 24–27 M parameters, also perform below the compact M4 model, significantly so for DeepLabV3+ (p=0.013) and PSPNet (p=0.0013). The relationship between mIoU and parameter count is shown in Figure 3.

The comparison also reveals differences between the evaluated encoder–decoder configurations. The two U-Net variants and the two SegFormer models achieve mIoU values between 87.70% and 88.85%, whereas DeepLabV3+ and PSPNet obtain lower values of 85.51% and 81.70%, respectively. This difference is particularly relevant for the small and thin plant structures present in CWFID. The qualitative predictions in Section 3.1.4 show that DeepLabV3+ and PSPNet more frequently fragment thin stems and misclassify small crop regions, whereas the U-Net and SegFormer models generally preserve these structures more consistently. These observations indicate that the choice of encoder–decoder configuration has a substantial effect on segmentation performance for this dataset.

#### 3.1.2. Per-Class Performance

**Figure 4:**
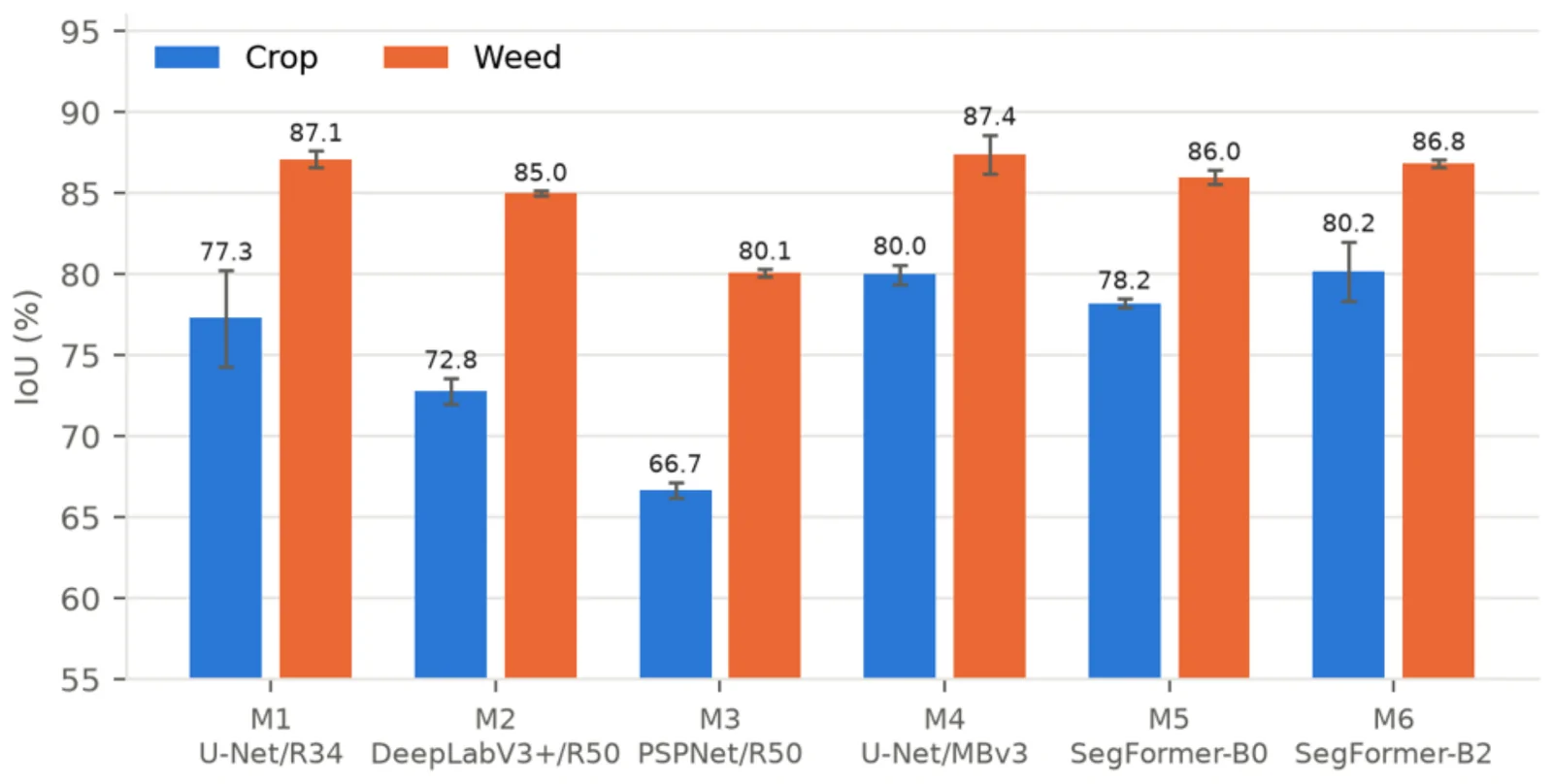
Per-class IoU for the crop and weed classes across the six dense segmentation models. Soil is omitted because its IoU exceeds 98% for all models.

A consistent limitation across all six architectures is the crop class. Crop IoU ranges from 66.67% for PSPNet to 80.19% for SegFormer-B2, whereas soil IoU remains between 98.36% and 99.14% and weed IoU between 80.08% and 87.43%, as shown in Figure 4. Consequently, the aggregate mIoU is strongly influenced by the relatively easy soil class. The crop class is therefore the main source of segmentation error in the dense models. Seed-to-seed variability is also more pronounced for crop IoU in M1 (±2.98) and M6 (±1.83), despite comparatively small variation in their aggregate mIoU values.

#### 3.1.3. Why Weed Is Easier than Crop

We identify three factors that explain the consistently higher weed IoU compared with crop IoU across all architectures. First, class imbalance contributes to the difference. As discussed in Section 2.1, weed accounts for 5.7% of the test pixels, compared with only 1.7% for crop. Consequently, the test set contains approximately 3.4 times more weed pixels than crop pixels, making a comparable number of classification errors proportionally more detrimental to crop IoU. Second, morphological differences between the classes make crop segmentation more challenging. Carrot seedlings have thin, feathery leaves, resulting in a larger proportion of boundary pixels, which are generally more difficult to classify, whereas the broader-leaved weed rosettes provide more spatially coherent regions. Third, the class confusion is asymmetric, as shown in Table 5, with one class being more frequently misclassified as the other. For M4, 7.0% of crop pixels are misclassified as weed and a further 4.5% as soil, whereas only 1.0% of weed pixels are misclassified as crop. Such crop-to-weed errors reduce crop IoU through both false negatives for the crop class and false positives for the weed class, while the corresponding weed-to-crop errors are comparatively rare. The same asymmetric pattern is observed across all six models and is particularly pronounced for the coarse-decoder architectures M2 and M3, for which 14–15% of crop pixels are misclassified as soil.

#### 3.1.4. Qualitative Results

**Figure 5:**
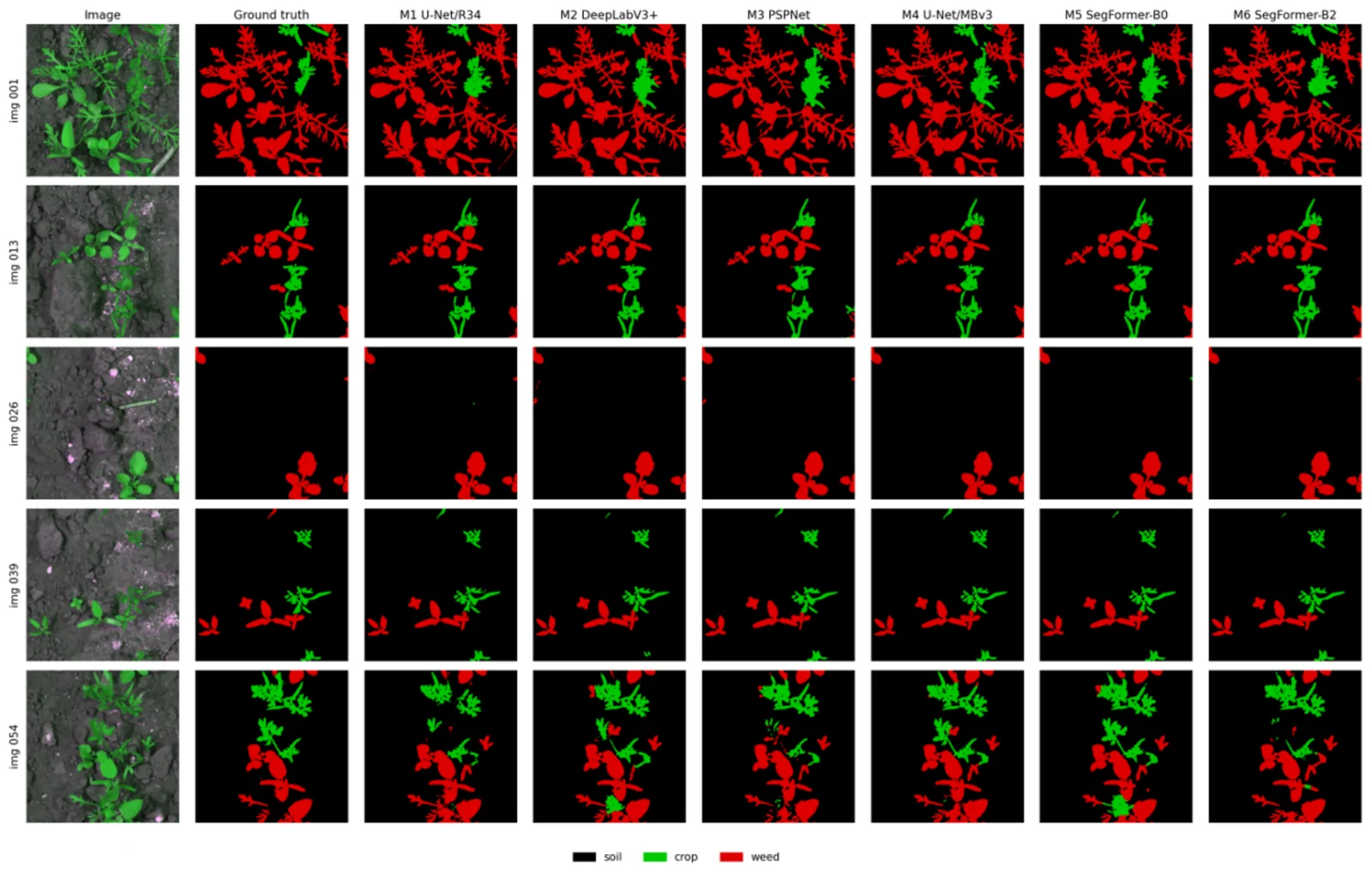
Qualitative comparison of the six dense segmentation models on five CWFID test images. Black, green, and red denote soil, crop, and weed, respectively.

**Figure 6:**
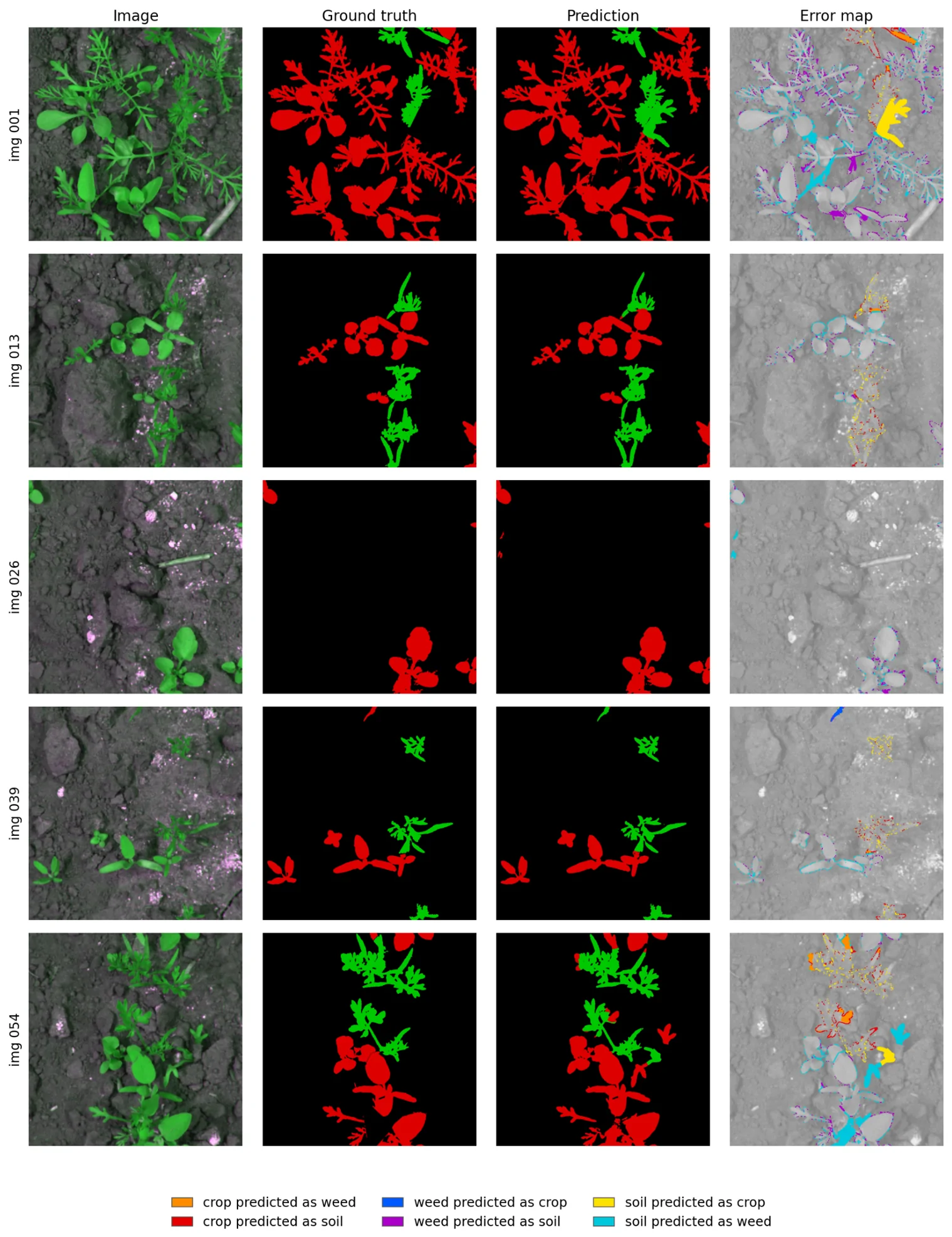
Error maps of the dense M4 model on five test images, overlaid on a greyscale copy of the field image. Each of the six possible confusions between the three classes has its own colour: orange, crop predicted as weed; red, crop predicted as soil; blue, weed predicted as crop; purple, weed predicted as soil; yellow, soil predicted as crop; cyan, soil predicted as weed. Error regions are dilated by one pixel for visibility.

Figure 5 provides qualitative predictions for five representative test images. All models recover the dominant soil regions and large weed structures reasonably well, while differences are more apparent around thin stems, small seedlings, and partially occluded crop–weed regions. PSPNet and DeepLabV3+ show more fragmented plant structures, whereas the U-Net and SegFormer configurations generally preserve connected crop regions more effectively. M4 provides visually consistent crop masks across the examples, including scenes containing dense mixtures of carrot leaves and weeds. Figure 6 makes the error structure of M4 explicit by assigning a distinct colour to each of the six possible confusions between soil, crop and weed. Errors concentrate at three locations. First, along thin stems and leaf boundaries, where they appear as one-pixel-wide contours of weed predicted as soil (purple) or soil predicted as weed (cyan) around the weed rosettes, and of crop predicted as soil (red) or soil predicted as crop (yellow) around the carrot foliage; these boundary errors are the dominant error type in every image. Second, on small or partially occluded carrot seedlings, which are predicted as weed (orange), as in images 013 and 054. Third, on crop leaves inside dense crop–weed mixtures (image 054), where orange and red regions extend over entire leaflets rather than only along edges. The reverse confusion, weed predicted as crop (blue), is rare and confined to a single small region in image 039, which is consistent with the asymmetry of the confusion matrices in Table 5. The error maps also reveal that several large yellow and cyan regions in images 001 and 054 are plants that are clearly visible in the photograph but absent from the ground-truth annotation; a part of the apparent false-positive rate for the plant classes therefore reflects annotation incompleteness in CWFID rather than model error.

#### 3.1.5. Model Selection

Considering both segmentation accuracy and model compactness, M4 is selected as the reference model for the subsequent pruning experiments. Its accuracy is not statistically distinguishable from that of the best transformer model while using 3.7× fewer parameters, is the second-smallest network in the study, and, being purely convolutional, permits structured pruning of every layer.

### 3.2. Pruning Trade-Offs on M4

**Figure 7:**
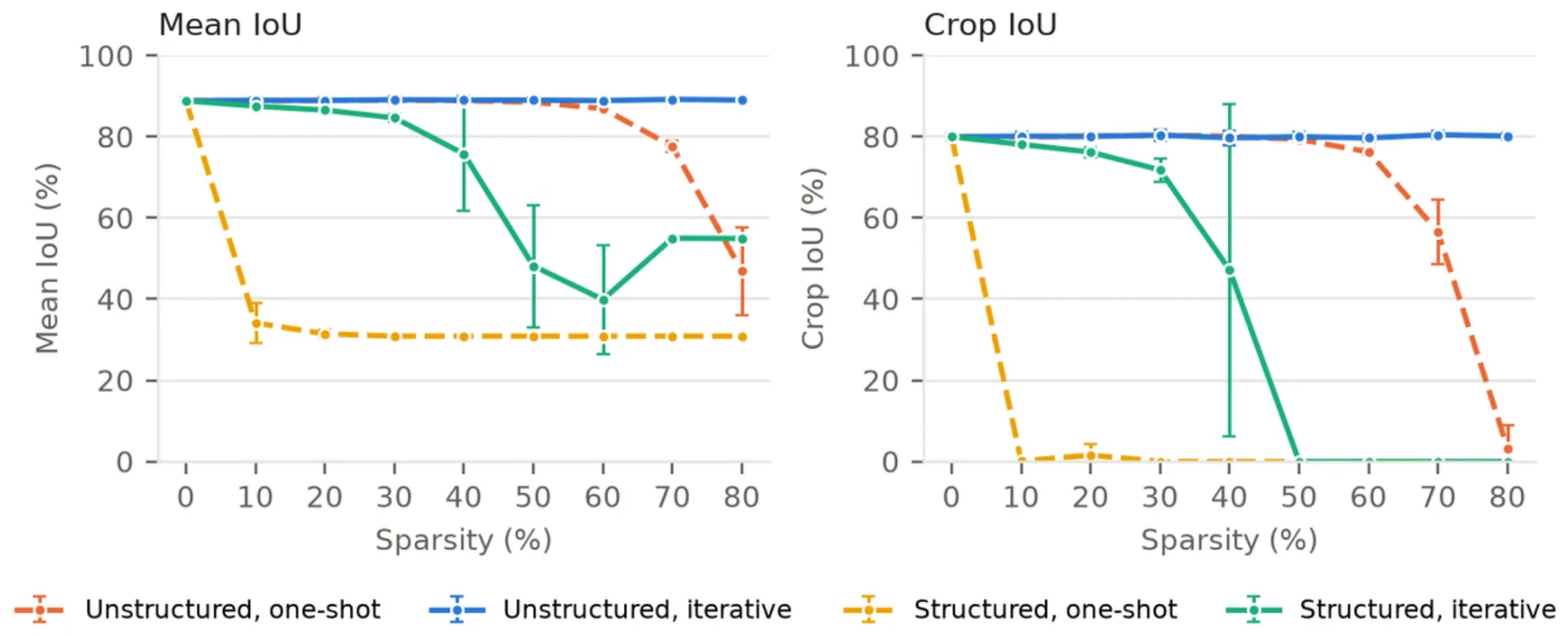
Effect of pruning sparsity on M4. (**Left**) mIoU; (**right**) crop IoU. Results are shown for one-shot and iterative unstructured pruning and for one-shot and iterative structured pruning. Error bars indicate one standard deviation over three seeds.

Table 6 reports the mIoU of M4 under the four pruning regimes at eight sparsity levels, Table 7 reports the corresponding computational characteristics, and Figure 7 reports the resulting mIoU and crop IoU curves; per-class IoU for all three classes is given in Section 3.4. The two pruning modes behave in opposite ways, and we describe them in turn.

#### 3.2.1. Unstructured Pruning

One-shot removal of low-magnitude weights produces only a small accuracy loss through 50% sparsity. At 50%, mIoU decreases from 88.85% for the dense model to 88.50%, a reduction of only 0.35 percentage points. The degradation becomes progressively larger at higher sparsities, reaching 86.94% at 60%, 77.60% at 70%, and 46.88% at 80%, where the large standard deviation (±10.73) indicates substantial sensitivity to the training seed. The iterative unstructured schedule is substantially more robust. After progressive pruning and fine-tuning, mIoU remains between 88.90% and 89.21% across all pruned sparsity levels, and the corresponding curve in Figure 7 is essentially flat. At 80% sparsity, the model retains 89.04±0.14% mIoU and 80.16±0.85% crop IoU while only 1.36 M of the original 6.69 M weights remain non-zero, as shown in Table 7. The small positive differences from the dense baseline at several sparsity levels should not be interpreted as an accuracy improvement caused by pruning; they are more appropriately attributed to training variation and the additional fine-tuning performed during the iterative schedule.

#### 3.2.2. Sparsity vs. Physical Compression

The computational implications of unstructured pruning require a careful distinction between sparsity and physical model reduction. As Table 7 shows, although 80% of the weights are zero, the dense parameter count, GFLOPs, and checkpoint size remain unchanged at every sparsity level in the implementation used here; only the non-zero weight count decreases. Consequently, unstructured pruning primarily reduces the number of stored non-zero weights and can provide computational benefits only when the deployment hardware and inference software exploit sparse representations or sparse kernels. It should therefore not be interpreted as a direct 80% reduction in dense inference cost.

#### 3.2.3. Structured Pruning

Structured channel pruning produces a different trade-off. Unlike unstructured pruning, removing channels reduces the actual parameter count, GFLOPs, and the serialized model as shown in Table 7. However, one-shot structured pruning is highly sensitive to even modest channel removal. At 10% sparsity, mIoU falls from 88.85% to 34.13±4.89%, and at 20% and beyond, the one-shot models obtain 30.9–31.4% mIoU, corresponding to the constant-soil prediction observed in the experiments and visible as the flat line near 31% in Figure 7. Iterative structured pruning substantially improves robustness at low sparsity levels. With progressive pruning and fine-tuning, the 10%, 20%, and 30% models reach 87.55%, 86.56%, and 84.64% mIoU, respectively. At 30% channel sparsity, the model contains 3.27 M parameters, requires 11.6 GFLOPs, and occupies 13.5 MB, corresponding to approximately a 2.0× reduction in parameters and a 2.1× reduction in GFLOPs relative to the dense M4 model. This operating point therefore provides a substantial reduction in computational cost while retaining comparatively strong segmentation performance. Beyond 30% sparsity, structured pruning becomes increasingly unstable. At 40%, the iterative models achieve 75.78±14.09% mIoU, reflecting a large difference among the three seeds. At 50% and 60%, mean mIoU falls to 48.16% and 39.89%, respectively. At 70% and 80%, the mean mIoU increases to approximately 55%, but these values do not represent recovery of the complete segmentation task, because the crop IoU in the right panel of Figure 7 remains zero: the predictions at these sparsities recover soil and weed regions but fail to recover the crop class.

#### 3.2.4. Measured Inference Latency

To examine whether the reductions in model size and computational cost translate into faster inference, Table 8 reports the measured latency of the dense and pruned M4 models on the training workstation. All measurements were obtained using PyTorch 2.14.1+cu130 eager execution with a batch size of 1, a 512×512 input, and FP32 precision, with the laptop connected to mains power. For the GPU, the reported values are the median and 95th-percentile latency over 100 timed iterations after 20 warm-up iterations. For the CPU, the same statistics were computed over 20 timed iterations after 3 warm-up iterations.

On the RTX 5080 Laptop GPU, structured pruning produces only small changes in inference latency. The dense model has a median latency of 5.11 ms, while the 10%, 20%, and 30% structured-pruned models achieve 5.43, 5.01, and 4.92 ms, respectively. Thus, the 30% structured-pruned model is only about 3.7% faster than the dense model despite reducing the parameter count from 6.69 M to 3.27 M. The 80% unstructured-pruned model also shows a similar latency to the dense model (5.07 ms). These results indicate that the reduction in parameters and GFLOPs does not translate into a proportional reduction in GPU inference time for this relatively small model.

The effect of structured pruning is more evident on the Intel Core Ultra 9 CPU. The dense model requires 51.4 ms per tile, whereas the 20% and 30% structured-pruned models reduce the latency to 42.1 and 38.4 ms, corresponding to reductions of approximately 18.1% and 25.3%, respectively. The 10% structured-pruned model, however, is slightly slower than the dense model (54.9 ms). In contrast, the 80% unstructured-pruned model also provides no latency improvement, with a median latency of 52.0 ms. This suggests that structured pruning can provide practical CPU inference benefits when it reduces the dimensions of the computations, whereas unstructured sparsity does not necessarily accelerate standard dense inference.

Overall, the latency results show that reductions in parameters and GFLOPs reported in Table 7 do not necessarily result in proportional reductions in wall-clock inference time. The realized speed-up depends on the pruning strategy and the target hardware and runtime. In this experiment, structured pruning provides limited latency improvement on the GPU but a more noticeable reduction in CPU inference time at 20–30% sparsity.

### 3.3. Effect of Pruning Schedule and Fine-Tuning

The comparison between one-shot and iterative pruning in Table 6 demonstrates the importance of the pruning schedule. For unstructured pruning, the difference becomes particularly pronounced at high sparsity: at 80%, one-shot pruning reduces mIoU to 46.88%, whereas iterative pruning maintains 89.04%. For structured pruning, one-shot removal fails at the first 10% level, while the iterative schedule retains 87.55% mIoU at the same sparsity and remains within 4.2 points of the dense model up to 30% (Section 3.2). Thus, gradual removal of channels followed by recovery training allows the network to adapt to the reduced representation capacity. Nevertheless, fine-tuning does not fully compensate for aggressive structural compression: performance becomes unstable at 40% and collapses for the more aggressive settings under the evaluated training budget of 10 epochs per level.

#### Role of the Fine-Tuning Budget

The observed degradation of structured pruning beyond 30% should be interpreted in the context of the fixed recovery budget of 10 fine-tuning epochs per pruning level. This budget was selected a priori to provide the same recovery effort across all pruning levels and pruning strategies, thereby maintaining a consistent basis for comparison. Two observations help delimit the interpretation of this result. First, the same 10-epoch budget is sufficient to recover performance across all unstructured-pruning levels and for structured pruning up to 30%, indicating that the budget is not generally insufficient for the network under the experimental setting. Second, at 40% structured pruning, one of the three seeds exhibits a substantial performance collapse, whereas the other two remain at approximately 84% mIoU. This variability suggests that 40% represents a transition region in which recovery becomes sensitive to initialization and the available fine-tuning budget. A larger recovery budget or finer pruning increments may alter the observed transition point; however, these possibilities are not evaluated in the present study. In particular, whether the severe degradation observed at 50–80% structured pruning can be recovered through substantially longer fine-tuning remains an open question and is therefore identified as a limitation of the current experimental design.

The results therefore show that the pruning schedule is a major determinant of compression robustness. One-shot pruning can produce severe performance degradation even at relatively low structured sparsity, whereas progressive pruning with fine-tuning substantially extends the range over which the model remains usable.

### 3.4. Class-Wise and Qualitative Effects of Compression

#### 3.4.1. Class-Wise Effects

Table 9 reports soil, crop, and weed IoU at every sparsity level of both iterative schedules. The class-wise results indicate that aggregate mIoU can conceal important failures in crop segmentation. Under iterative structured pruning, crop IoU decreases from 80.0% for the dense model to 78.1%, 76.2%, and 71.8% at 10%, 20%, and 30% sparsity, respectively, whereas weed IoU decreases only from 87.4% to 83.3% and soil IoU from 99.1% to 98.8% over the same range. At 30% sparsity, this corresponds to an 8.2-point reduction in crop IoU, while the overall mIoU decreases by 4.21 points. At 40%, crop IoU drops to 47.2±40.9 while weed IoU remains at 81.8±0.7, and at 50% sparsity and above, crop IoU becomes zero while weed IoU is still 24–66% and soil IoU above 95%. In contrast, the crop IoU of the iteratively pruned unstructured models remains between 79.7% and 80.5% throughout the evaluated sparsity range, and no class changes by more than 0.8 IoU points at any level. Crop segmentation can also deteriorate before the overall mIoU exhibits a comparable reduction: at 70% one-shot unstructured sparsity, crop IoU falls to 56.5%, whereas weed IoU remains at 78.1%. This difference illustrates why crop-specific performance should be monitored when evaluating compression for selective agricultural operations rather than relying solely on aggregate mIoU. Regarding the transfer of confusion between classes, the error maps of the 30% structured model (Section 3.1.4) show that crop pixels lost under structured pruning are predominantly reassigned to the weed class rather than to soil: the pruned network continues to detect vegetation but progressively loses the ability to discriminate the two plant classes.

#### 3.4.2. Qualitative Results

**Figure 8:**
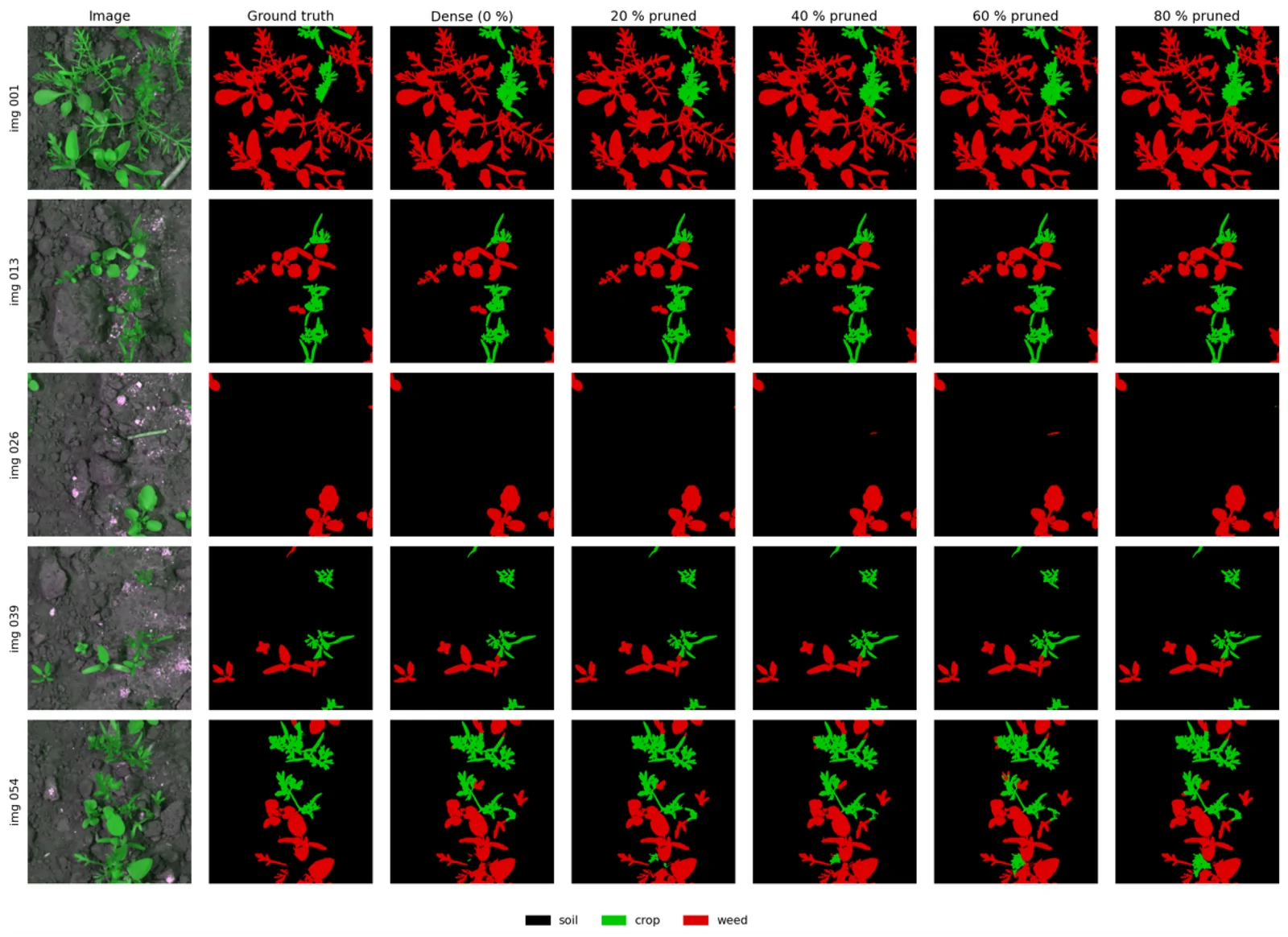
Qualitative predictions of M4 after iterative unstructured pruning at 20%, 40%, 60%, and 80% weight sparsity.

**Figure 9:**
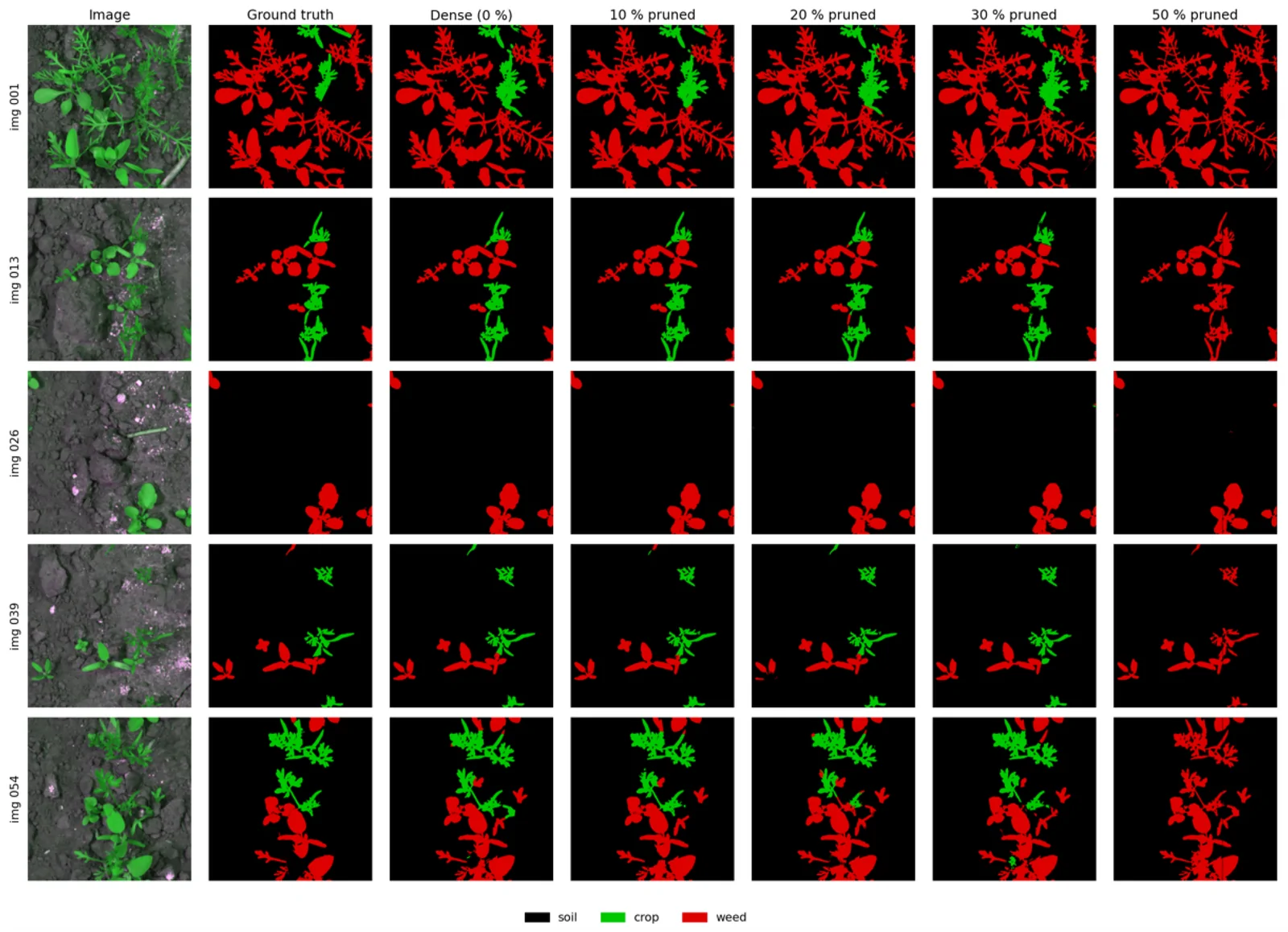
Qualitative predictions of M4 after iterative structured channel pruning at 10%, 20%, 30%, and 50% sparsity. At 50%, the model no longer predicts the crop class in the displayed examples.

Figure 8 and Figure 9 provide qualitative comparisons using the same five test images as the dense-model evaluation. The iteratively pruned unstructured models preserve visually similar segmentation masks from 20% through 80% weight sparsity. Thin stems remain connected and small carrot seedlings remain identifiable, with only minor boundary differences at the highest sparsity level.

The structured-pruning predictions show a different progression. At 10%, the segmentation remains visually close to the dense model. At 20%, thin crop leaves begin to be confused with weed, while at 30% the crop regions show visible erosion and relabelling. These qualitative changes are consistent with the reduction in crop IoU reported in Table 9. At 50%, the network no longer predicts the crop class, and detected plant pixels are predominantly assigned to the weed class.

**Figure 10:**
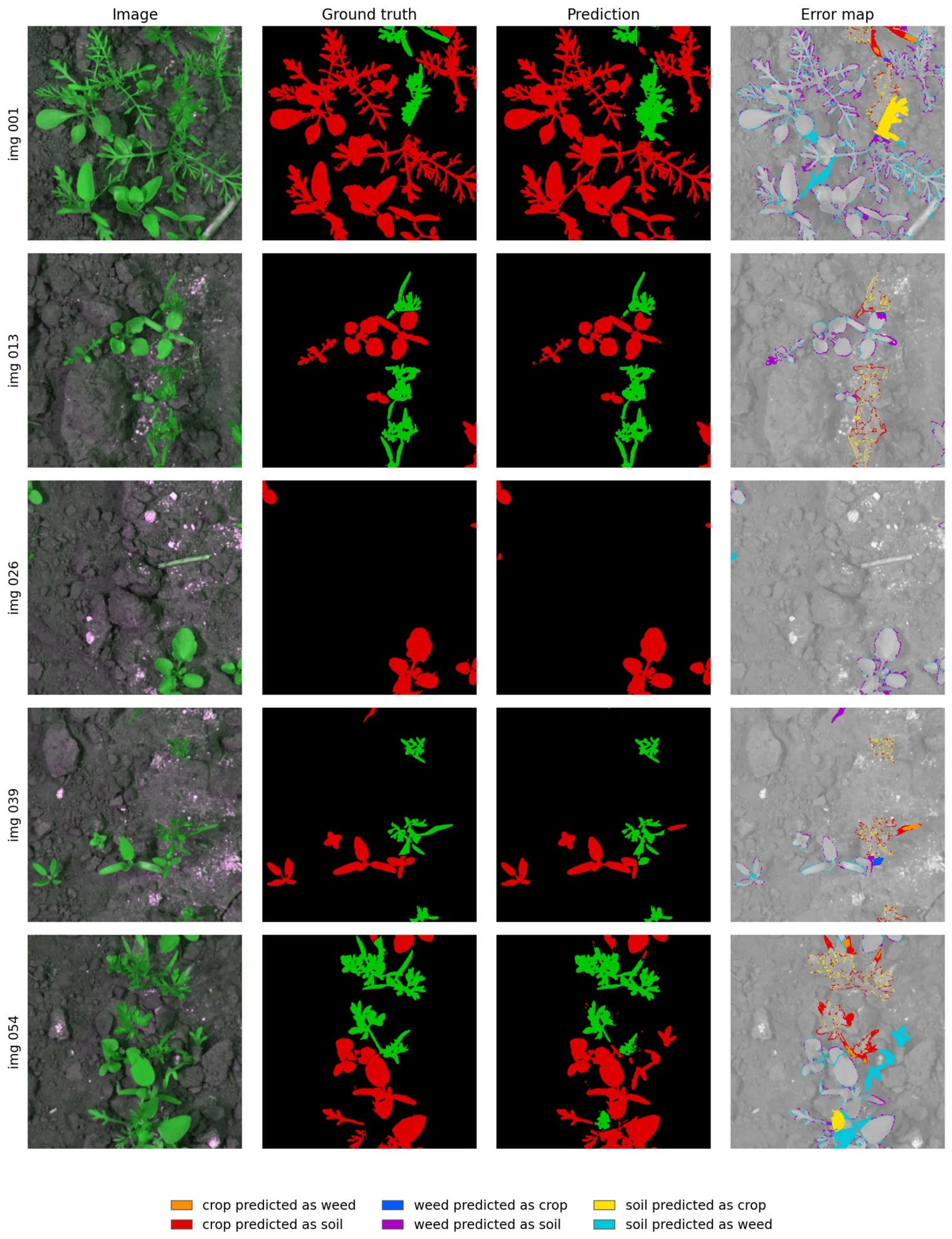
Error maps of M4 after 30% iterative structured pruning on the five test images. Each of the six possible confusions between the three classes has its own colour: orange, crop predicted as weed; red, crop predicted as soil; blue, weed predicted as crop; purple, weed predicted as soil; yellow, soil predicted as crop; cyan, soil predicted as weed. Error regions are dilated by one pixel for visibility.

The error maps in Figure 10 show that the additional errors introduced by 30% structured pruning appear at the same locations as those of the dense model, thin structures and crop–weed boundaries, but cover larger areas, and that they fall predominantly on the crop class. Around the weed rosettes, the boundary contours of weed predicted as soil (purple) and soil predicted as weed (cyan) are only slightly thicker than in Figure 6, whereas around the carrot foliage the contours of crop predicted as soil (red) and soil predicted as crop (yellow) have grown from thin outlines into filled regions that cover entire leaflets (images 013, 039 and 054). Crop predicted as weed (orange) likewise expands from isolated leaf tips in the dense model to complete leaflets in image 039 and to a whole seedling in image 054. Weed predicted as crop (blue) remains rare. The pruned model therefore still separates vegetation from soil about as well as the dense model, but progressively loses the finer distinction between the two plant classes, with the loss borne almost entirely by crop; this is the spatial counterpart of the 8.2-point drop in crop IoU against 4.1 points for weed at this sparsity level (Table 9).

Overall, the experimental results establish three main findings. First, the compact U-Net/MobileNetV3-L configuration provides the strongest dense accuracy–efficiency balance among the evaluated models. Second, unstructured pruning is highly tolerant to aggressive sparsity when introduced progressively, although it does not directly reduce dense computational cost or measured latency. Third, structured pruning provides genuine reductions in parameters, GFLOPs, and model size, but its usable sparsity range is substantially narrower; under the evaluated protocol, 30% channel sparsity represents the highest tested level that retains comparatively robust segmentation performance. Across the compression experiments, crop segmentation is also more sensitive than the aggregate mIoU alone suggests, emphasizing the need for class-wise evaluation in agricultural edge-deployment scenarios.

## 4. Discussion

This study systematically examined the accuracy–efficiency trade-offs of six semantic segmentation architectures and evaluated how unstructured and structured pruning affect segmentation performance and model complexity under a common experimental protocol. The findings provide evidence for the three research questions and highlight an important distinction between numerical sparsity and physically deployable model compression.

### 4.1. Architecture and Compression Trade-Offs

**RQ1: Which segmentation architecture provides the most favorable accuracy–efficiency trade-off for crop–weed segmentation under a common training and evaluation protocol?**

Among the evaluated architectures, U-Net with a MobileNetV3-Large encoder (M4) provided the most favorable balance between segmentation accuracy and model complexity. It achieved 88.85±0.46% mIoU, a value from which SegFormer-B2 could not be statistically separated with three seeds (*p* = 0.58), while requiring substantially fewer parameters than the ResNet-based models and SegFormer-B2, and fewer GFLOPs than all of them except PSPNet, whose pyramid-pooling decoder is computationally cheap but markedly less accurate. SegFormer-B0 achieved a similar mIoU with an even smaller parameter count, but M4 provided the best combination of accuracy and structured-pruning suitability among the evaluated models. The results therefore suggest that greater model capacity does not necessarily translate into better segmentation performance under the same training and evaluation conditions. Given three seeds, however, only the differences between M4 and the coarse-decoder models are statistically established; the ordering among M4, M6, M1, and M5 should be read as a tie.

The class-wise results further indicate that the main challenge is distinguishing crop from visually similar plant structures rather than identifying soil. Soil IoU exceeded 98% for all models, whereas crop IoU was consistently lower. The confusion matrices and error maps attribute this to class imbalance (3.4 times fewer crop than weed pixels), to the thin morphology of carrot foliage, and to an asymmetric confusion in which crop pixels are lost to the weed class far more often than the reverse. This also explains why differences in aggregate mIoU should be interpreted together with class-specific performance. M4 was consequently selected as the reference architecture for compression because it combined strong segmentation performance with a relatively compact convolutional structure suitable for structured pruning. This conclusion is limited to the six architectures and common protocol evaluated here and should not be interpreted as a universal ranking of segmentation architectures.

**RQ2: How do unstructured weight pruning and structured channel pruning affect segmentation accuracy and computational complexity as sparsity increases?**

The two pruning strategies exhibited substantially different trade-offs. Iterative unstructured pruning preserved segmentation performance even at high sparsity, with mIoU remaining close to the dense baseline through 80% sparsity. However, this sparsity did not produce a corresponding reduction in dense parameter dimensions, GFLOPs, or checkpoint size in the evaluated implementation, nor in measured latency on either the GPU or the CPU (Table 8). Its practical deployment benefit therefore depends on hardware and software capable of exploiting sparse computation.

Structured pruning produced the opposite behavior. Removing channels directly reduced parameters, GFLOPs, and checkpoint size, but segmentation performance became increasingly sensitive as sparsity increased. Iterative structured pruning retained comparatively strong performance up to 30% sparsity, where the model was reduced from 6.69 M to 3.27 M parameters and from 24.7 to 11.6 GFLOPs. Beyond this point, performance degraded substantially. These findings demonstrate that sparsity alone is insufficient for evaluating compression: unstructured pruning can achieve high numerical sparsity without physical model reduction, whereas structured pruning provides direct architectural compression but imposes a stronger constraint on the learned representation. The latency measurements add a further caveat: even the physical FLOP reduction of structured pruning translates into wall-clock savings only where the network is arithmetic-bound, as on the CPU (25% at 30% sparsity), and hardly at all on a discrete laptop GPU (3.7%), where a network of this size is launch-bound at batch size 1.

### 4.2. Effect of Pruning Schedule and Class Degradation

**RQ3: How do pruning schedules and fine-tuning influence compression robustness, and which semantic classes degrade first under increasingly aggressive pruning?**

The comparison between one-shot and iterative pruning demonstrates that the pruning schedule is a major determinant of compression robustness. One-shot pruning caused severe performance degradation, particularly for structured pruning, whereas progressive pruning followed by fine-tuning substantially improved recovery. For example, at 10% structured sparsity, one-shot pruning reduced mIoU to approximately 34%, while iterative pruning retained approximately 88%. Similarly, at 80% unstructured sparsity, iterative pruning maintained approximately 89% mIoU compared with approximately 47% for one-shot pruning. These results indicate that gradual parameter or channel removal allows the network to adapt to the reduced representation capacity more effectively than abrupt removal. This conclusion must be qualified by the design of the two arms: because the iterative schedule fine-tunes after every step, its advantage over one-shot pruning combines the benefit of gradual removal with that of up to 80 additional training epochs, and the present experiments do not separate the two. What the comparison establishes is that pruning without any recovery training is not viable for channel removal even at 10% and degrades weight pruning beyond 50%, and that the progressive prune-and-fine-tune protocol recovers this loss; whether the same recovery could be obtained by one-shot pruning followed by an equal fine-tuning budget is an open question.

The class-wise results also show that crop segmentation is more sensitive to aggressive structured compression than the aggregate mIoU suggests. Under iterative structured pruning, crop IoU decreased progressively from the dense baseline and reached zero at 50% sparsity and above, while weed and soil IoU degraded far less (Table 9). In contrast, crop IoU remained close to the dense value under iterative unstructured pruning throughout the evaluated sparsity range. The increase in aggregate mIoU observed at 70–80% structured sparsity should therefore not be interpreted as recovery of the original segmentation capability because the crop class remained absent. This finding highlights the importance of class-wise evaluation when assessing compression for agricultural applications, where preserving the crop class may be more relevant than maintaining aggregate performance alone.

### 4.3. Implications for Deployment

The results suggest that the appropriate compression strategy depends on the intended deployment objective. Iterative unstructured pruning is attractive when the deployment platform can exploit sparse weights and when preservation of segmentation accuracy is the primary objective. Structured pruning is more suitable when actual reductions in model dimensions, GFLOPs, and storage are required. Under the evaluated protocol, 30% iterative structured sparsity provides a practical compromise between physical compression and segmentation performance, whereas higher sparsity levels involve substantial degradation, particularly for crop segmentation. As shown in Table 8, the 30% structured-pruned model reduces latency by approximately 25% on the CPU (from 51.4 to 38.4 ms per tile), but by only 3.7% on the RTX 5080 Laptop GPU (from 5.11 to 4.92 ms). Thus, the 2.1× reduction in GFLOPs translates only partially into wall-clock speed-up, highlighting the dependence of practical performance on the target hardware and inference runtime. These results also show that latency and energy benefits should be evaluated directly on the hardware on which the model is intended to operate.

### 4.4. Limitations

Several limitations should be considered when interpreting the findings as follows:

**Single dataset.** All experiments were conducted on CWFID, which contains images of carrot fields acquired under a specific field and imaging setup. Consequently, the absolute accuracies, relative architecture performance, and class-wise behavior observed in this study may differ for other crops, growth stages, field conditions, illumination settings, or imaging devices. The use of a single dataset was intentional because the primary objective was a controlled comparison in which all architectures and pruning regimes were evaluated using the same data, preprocessing, training protocol, and test procedure. Nevertheless, this design limits the external validity of the reported accuracy values and compression operating points. The findings should therefore be interpreted as evidence of the behavior of the evaluated methods on this particular crop–weed segmentation benchmark rather than as universal performance estimates. The general distinction between unstructured sparsity and physical structured compression is conceptually broader, but its quantitative effect remains dataset- and task-dependent.

**Single pruned architecture.** The pruning experiments were performed only on M4, which was selected based on its combination of segmentation performance and model compactness among the six dense architectures. This choice provides a consistent basis for comparing pruning strategies but limits the extent to which the pruning results can be generalized to other architectures. In particular, structured-pruning sensitivity can depend on layer width, block design, skip connections, and channel dependencies. The MobileNetV3-Large architecture used by M4 contains relatively narrow layers, which may contribute to its sensitivity to channel removal. Therefore, the observed structured-pruning behavior, including the approximately 30% operating point identified in this study, should be regarded as specific to M4 and the evaluated pruning procedure rather than as a general limit for structured pruning.

**Pruning protocol.** The structured-pruning results depend on the selected pruning criterion, channel-dependency procedure, pruning schedule, and fine-tuning budget. The study used L1-based channel importance, progressive pruning, and a fixed recovery budget of 10 epochs per pruning level to provide a consistent and reproducible baseline. Alternative strategies, including layer-sensitive pruning, gradient- or Taylor-based importance measures, batch-normalization recalibration, smaller pruning increments, longer recovery training, or hardware-aware channel constraints, were not investigated. Consequently, the observed degradation beyond 30% structured sparsity represents the behavior of the evaluated protocol and should not be interpreted as the maximum achievable compression for M4.

**Statistical power and deployment metrics.** Each experiment was repeated with three random seeds, which provides an estimate of training variability but offers limited statistical evidence for resolving small differences between configurations. The results should therefore be interpreted primarily in terms of the observed effect sizes and variability rather than small differences in mean performance. In addition, computational efficiency was characterized using parameter count, GFLOPs, checkpoint size, and inference latency. Latency was measured only on the training workstation (a laptop GPU and a desktop-class CPU, PyTorch eager, FP32), not on an embedded agricultural platform, and memory use, energy consumption, and throughput on such devices were not measured. The practical benefit of unstructured sparsity also depends on whether the target hardware and inference software can exploit sparse computation. Validation on representative edge hardware is therefore required before the reported reductions can be translated into deployment-time performance claims.

## 5. Conclusions

This study systematically evaluated segmentation architectures and pruning strategies for crop–weed segmentation on the CWFID dataset under a common training and evaluation protocol. Among the six dense models, U-Net with a MobileNetV3-Large encoder provided the most favorable accuracy–efficiency trade-off, achieving 88.85±0.46% mIoU with 6.69 M parameters and 24.7 GFLOPs, an accuracy from which SegFormer-B2 could not be statistically separated under the evaluated protocol, with 3.7× fewer parameters. This model was therefore selected as the reference architecture for the subsequent compression experiments.

The pruning experiments revealed distinct trade-offs between unstructured and structured pruning. Iterative unstructured pruning maintained segmentation performance up to 80% weight sparsity, achieving 89.04±0.14% mIoU with only 1.36 M non-zero weights. However, because the dense tensor dimensions were unchanged, this sparsity did not reduce the GFLOPs, serialized model size, or measured latency. In contrast, iterative structured channel pruning produced actual reductions in parameters, GFLOPs, and model size, but with a substantially narrower accuracy-preserving range. At 30% channel sparsity, the model was reduced to 3.27 M parameters and 11.6 GFLOPs while retaining 84.64±0.97% mIoU. Beyond this level, performance became increasingly unstable, with crop segmentation degrading substantially and eventually reaching zero IoU at higher sparsities.

The results further demonstrate that pruning schedule and fine-tuning are important for compression robustness. Progressive pruning with recovery training substantially outperformed one-shot pruning, particularly for structured compression. The class-wise analysis also showed that crop segmentation is more sensitive to aggressive compression than soil and weed and, in several cases, than the aggregate mIoU suggests. These findings indicate that compression evaluation for agricultural segmentation should consider both physical model reduction and class-specific performance rather than sparsity or overall mIoU alone.

Overall, the study provides an empirical reference for selecting segmentation architectures and compression strategies under a controlled experimental setting. The findings suggest that the appropriate pruning strategy depends on the intended deployment objective: unstructured pruning offers high sparsity with strong accuracy retention, whereas structured pruning offers more direct computational and model-size reductions but requires a more conservative compression level.

Several directions remain for future investigation. First, the compression strategies should be evaluated on physical edge and agricultural computing platforms to measure actual inference latency, memory consumption, energy use, and throughput. Second, future work could investigate hardware-aware and layer-wise structured pruning to determine whether the usable compression range can be extended while preserving crop segmentation. Third, the robustness of the observed trade-offs should be examined across additional crop–weed datasets, field conditions, lighting conditions, and acquisition devices. In particular, evaluating the models and pruning strategies on independent crop–weed datasets not used in this study would provide a direct assessment of their external generalizability. Finally, combining structured pruning with complementary compression techniques, such as quantization or knowledge distillation, may provide further reductions in deployment cost while maintaining segmentation quality.

## Figures and Tables

**Table 1:** Architectures and their complexity for a single 512×512 input image.

ID	Architecture	Encoder	Family	Params (M)	GFLOPs	Size (MB)
M1	U-Net	ResNet-34	CNN	24.44	62.9	98.0
M2	DeepLabV3+	ResNet-50	CNN	26.68	73.8	107.1
M3	PSPNet	ResNet-50	CNN	24.31	23.8	97.6
M4	U-Net	MobileNetV3-L	CNN	6.69	24.7	27.1
M5	SegFormer	MiT-B0	Trans.	3.71	13.5	15.0
M6	SegFormer	MiT-B2	Trans.	24.72	42.4	99.1

**Table 2:** Training and pruning configurations used in the experiments. The dense training protocol was applied uniformly to all architectures, while the fine-tuning protocol was applied after each step of iterative pruning.

Parameter	Dense Training	Iterative-Pruning Fine-Tuning
Optimizer	AdamW, weight decay $1\times 10^{-4}$	AdamW, weight decay $1\times 10^{-4}$
Learning rate	One-cycle, peak $3\times 10^{-4}$, 10% warm-up	One-cycle, peak $1\times 10^{-4}$, 10% warm-up
Loss	Cross-entropy + Dice	Cross-entropy + Dice
Epochs	60	10 per pruning step
Batch size	8	8
Precision	AMP (FP16/FP32)	AMP (FP16/FP32)
Input	512×512 tiles, stride 384	Same as dense
Augmentation	Flips, rotations, photometric	Same as dense
Random seeds	0, 1, 2	Inherited from dense model
Sparsity levels	–	10–80%, step 10%

**Table 3:** Dense segmentation baselines on the CWFID test split. Values are mean ± standard deviation over three seeds, reported in %. Best values in each column are shown in **bold**.

Model ID	mIoU	IoU_soil_	IoU_crop_	IoU_weed_	mF1
M1	87.80 ± 1.15	99.01 ± 0.04	77.28 ± 2.98	87.11 ± 0.49	93.26 ± 0.72
M2	85.51 ± 0.22	98.74 ± 0.02	72.78 ± 0.81	85.02 ± 0.13	91.84 ± 0.15
M3	81.70 ± 0.10	98.36 ± 0.02	66.67 ± 0.50	80.08 ± 0.24	89.37 ± 0.08
M4	**88.85 ± 0.46**	**99.14 ± 0.07**	80.00 ± 0.61	87.43 ± 1.19	**93.91 ± 0.26**
M5	87.70 ± 0.20	98.88 ± 0.02	78.21 ± 0.29	86.00 ± 0.45	93.23 ± 0.12
M6	88.67 ± 0.53	98.97 ± 0.02	**80.19 ± 1.83**	86.83 ± 0.23	93.81 ± 0.33

**Table 4:** Paired comparison of M4 with each of the other five architectures across the three seeds.

Comparison	ΔmIoU	SD of Δ	*t*	*p*
M4 vs. M6 (SegFormer-B2)	+0.19	0.50	0.65	0.58
M4 vs. M5 (SegFormer-B0)	+1.16	0.62	3.24	0.08
M4 vs. M1 (U-Net/R34)	+1.05	1.19	1.53	0.27
M4 vs. M2 (DeepLabV3+)	+3.34	0.66	8.78	0.013
M4 vs. M3 (PSPNet)	+7.15	0.44	28.0	0.0013

**Table 5:** Row-normalised confusion matrices of the six dense models on the test split (seed 0), in % of the pixels of each true class.

Model	True Crop →			True Weed →		
	Soil	Crop	Weed	Soil	Crop	Weed
M1	11.91	80.70	7.39	4.67	0.93	94.41
M2	14.37	79.25	6.39	6.28	0.40	93.32
M3	15.48	75.62	8.90	9.64	0.72	89.64
M4	4.52	88.50	6.98	4.02	0.99	94.98
M5	6.40	89.00	4.59	5.20	0.92	93.88
M6	7.92	86.95	5.13	4.46	0.84	94.69

**Table 6:** Mean IoU (%) of M4 (U-Net/MobileNetV3-L) under different pruning strategies and sparsity levels. Values are the mean ± standard deviation over three seeds.

Sparsity	Unstructured Pruning		Structured Pruning	
	One-Shot	Iterative	One-Shot	Iterative
0%	88.85±0.46	88.85±0.46	88.85±0.46	88.85±0.46
10%	88.85±0.44	88.97±0.78	34.13±4.89	87.55±0.11
20%	88.92±0.46	88.96±0.65	31.43±0.98	86.56±0.19
30%	89.03±0.35	89.12±0.79	30.87±0.00	84.64±0.97
40%	88.92±0.45	89.04±0.80	30.87±0.00	75.78±14.09
50%	88.50±0.26	89.01±0.61	30.87±0.00	48.16±15.10
60%	86.94±0.29	88.90±0.35	30.87±0.00	39.89±13.39
70%	77.60±1.38	89.21±0.27	30.87±0.00	54.99±0.04
80%	46.88±10.73	89.04±0.14	30.87±0.00	54.92±0.37

**Table 7:** Computational characteristics of M4 under increasing pruning sparsity (iterative schedule). The non-zero weight count applies to unstructured pruning, whose dense parameters, GFLOPs, and size remain at the 0% values; the remaining columns apply to structured pruning.

Sparsity	Unstructured Pruning	Structured Pruning		
	Non-Zero (M)	Params (M)	GFLOPs	Size (MB)
0%	6.69	6.69	24.7	27.1
10%	6.02	5.41	19.7	22.0
20%	5.36	4.27	15.3	17.5
30%	4.69	3.27	11.6	13.5
40%	4.02	2.41	8.4	10.0
50%	3.36	1.68	5.9	7.0
60%	2.69	1.08	3.7	4.6
70%	2.03	0.61	2.2	2.8
80%	1.36	0.27	1.0	1.4

**Table 8:** Measured inference latency of dense and pruned M4 on the training workstation (batch size 1, 512×512 input, FP32, PyTorch eager; laptop on mains power). Median and 95th percentile over 100 timed iterations on the GPU and 20 on the CPU, after 20 and 3 warm-up iterations respectively.

Device	Configuration	Params (M)	Median (ms)	p95 (ms)	FPS
RTX 5080 Laptop GPU	Dense	6.69	5.11	5.58	195.8
RTX 5080 Laptop GPU	Structured 10%	5.41	5.43	5.86	184.2
RTX 5080 Laptop GPU	Structured 20%	4.27	5.01	5.23	199.7
RTX 5080 Laptop GPU	Structured 30%	3.27	4.92	5.30	203.3
RTX 5080 Laptop GPU	Unstructured 80%	6.69	5.07	5.81	197.4
Intel Core Ultra 9 (CPU)	Dense	6.69	51.4	59.7	19.4
Intel Core Ultra 9 (CPU)	Structured 10%	5.41	54.9	63.5	18.2
Intel Core Ultra 9 (CPU)	Structured 20%	4.27	42.1	51.9	23.7
Intel Core Ultra 9 (CPU)	Structured 30%	3.27	38.4	43.9	26.0
Intel Core Ultra 9 (CPU)	Unstructured 80%	6.69	52.0	66.6	19.2

**Table 9:** Per-class IoU (%) of M4 under the two iterative pruning schedules, mean ± standard deviation over three seeds.

Sparsity	Unstructured, Iterative			Structured, Iterative		
	Soil	Crop	Weed	Soil	Crop	Weed
0%	99.1±0.1	80.0±0.6	87.4±1.2	99.1±0.1	80.0±0.6	87.4±1.2
10%	99.2±0.1	80.1±1.0	87.6±1.3	99.0±0.1	78.1±0.5	85.5±0.5
20%	99.2±0.1	80.1±0.6	87.7±1.3	99.0±0.1	76.2±1.2	84.5±0.7
30%	99.1±0.1	80.4±1.3	87.8±1.0	98.8±0.1	71.8±2.8	83.3±0.6
40%	99.2±0.0	79.7±1.7	88.2±0.7	98.4±0.8	47.2±40.9	81.8±0.7
50%	99.2±0.0	80.0±1.2	87.9±0.7	96.2±3.2	0.0±0.0	48.3±42.3
60%	99.2±0.0	79.7±0.8	87.8±0.7	95.1±3.2	0.0±0.0	24.6±37.0
70%	99.2±0.0	80.5±0.9	88.0±0.6	98.5±0.0	0.0±0.0	66.4±0.1
80%	99.1±0.0	80.2±0.8	87.8±0.4	98.5±0.1	0.0±0.0	66.2±1.0

## Data Availability

The data presented in this study are openly available in Crop/Weed Field Image Dataset (CWFID) analysed in this study is publicly available at https://github.com/cwfid/dataset (accessed on 28 August 2026).

## Abbreviations

The following abbreviations are used in this manuscript:

CNN	Convolutional Neural Network
CWFID	Crop/Weed Field Image Dataset
IoU	Intersection over Union
mIoU	Mean Intersection over Union
MiT	Mix Transformer
GFLOPs	Giga Floating-Point Operations
AMP	Automatic mixed precision
AdamW	Adaptive Moment Estimation with Weight Decay
FP16/32	Floating Point 16/32-bit
TP	True Positive
FP	False Positive
FN	False Negative
